# Vicarious Benevolence

**Published:** 1892-06-04

**Authors:** 


### PASSING TOPICS.

### VICARIOUS BENEVOLENCE.

We cannot profess admiration for the generosity which consists of giving away what belongs to somebody else. Even in that remote period when the century was young and the history of Sandford and Merton was accepted as a serious exposition of morals, Tommy was subjected to a sententious rebuke far beyond his years, for having fed the starving upon the Rector's loaves. Life is rich in instances of vicarious good nature, and one of the most recent forms of its development appears as questionable as any of its predecessors. Why should certain people write to the papers in order to suggest, first, that particular comforts should be taken from other people who pay for them and distributed to all comers, and secondly, to fasten the term "selfish" upon those who object to the process?

It has become quite the fashion with those who do not live in squares to upbraid those who do for not throwing open the enclosures to the "squalid inhabitants of the surrounding slums." The lawyers, for instance, who pay heavy rents for such quiet as Lincoln's Inn Fields afford are periodically brought to judgment upon *ex parte* statements and left for execution, because they do not see their way to turn their attractive glades into a playground for Clare Market and the purlieus of Drury Lane. With all the desire in the world to be philanthropic we fail to follow the reasoning. Occupiers who pay increased rents in return for certain advantages of neighbourhood and surroundings have as much right to enjoy what they pay for as anybody else. Very often there is a specific assessment for the square, and those who contribute to maintain open spaces in the heart of London assist, unintentionally it may be, but none the less surely, in benefiting the locality they live in.

We are fully alive to the duties of property, but unless it is proposed that men should hold all things in common, there can be no moral claim upon one class more than another to forego the advantages of which they stand rightfully possessed. This is no question of rich *versus* poor. To not a few inhabitants of squares the quiet and retirement are less luxuries than helps to work, and harder work, too, than mere hand toilers ever dream of. When the dwellers in squares, whether Belgravian or less select, are upbraided for not willingly consenting to have their nooks turned into bear gardens, have their critics considered all that is logically included in the demand and are they prepared to abide the consequences? The argument that because the inhabitants seldom promenade in the enclosure they will not be prejudiced if it be invaded by all sorts and conditions of men is capable of application to many other comforts and luxuries. If the front garden is to be appropriated, it will be only a question of time when the back garden must go too, and equally convincing claims might be advanced in reference to the spare bed-room.

As a matter of fact, the benefits of the parks and public gardens are already in a large degree monopolised by the loafing and dirty, as distinguished from the industrious and cleanly, classes. There seems no help for this, but we may reasonably regret that the advantages are not greater. It has been roundly asserted that the most constant *habitués* of our parks are apt to leave palpably unpleasant traces behind them, but, unhappily, this makes no appreciable difference in what they carry away. Whatever may be affirmed of Lincoln's Inn Fields in the vacation time, we suspect that anything like a serious effort at confiscating to the mob the quietude and beauty of our residential squares, which are at present an ornament and a blessing to the town, will be to invite a public misfortune, and possibly to hasten the advent of Macaulay's New Zealander. Agricola.

